# Just-in-Time Prompts for Running, Walking, and Performing Strength Exercises in the Built Environment: 4-Week Randomized Feasibility Study

**DOI:** 10.2196/35268

**Published:** 2022-08-01

**Authors:** Karlijn Sporrel, Shihan Wang, Dick D F Ettema, Nicky Nibbeling, Ben J A Krose, Marije Deutekom, Rémi D D de Boer, Monique Simons

**Affiliations:** 1 Human Geography and Spatial Planning Utrecht University Utrecht Netherlands; 2 Department of Information and Computing Sciences Utrecht University Utrecht Netherlands; 3 Informatics Institute University of Amsterdam Amsterdam Netherlands; 4 Faculty of Sports and Nutrition Amsterdam University of Applied Sciences Amsterdam Netherlands; 5 Department of Software Engineering University of Applied Sciences Amsterdam Amsterdam Netherlands; 6 Department of Health, Sports and Welfare Inholland University Haarlem Netherlands; 7 Consumption and Healthy Lifestyles group Wageningen University & Research, Wageningen Wageningen Netherlands

**Keywords:** just-in-time interventions, context-based, prompts, reminders, physical activity, mobile health, mHealth, exercise application, Fogg Behavior Model, user experience, engagement, feasibility study, mobile phone

## Abstract

**Background:**

App-based mobile health exercise interventions can motivate individuals to engage in more physical activity (PA). According to the Fogg Behavior Model, it is important that the individual receive prompts at the *right* time to be successfully persuaded into PA. These are referred to as *just-in-time* (JIT) *interventions*. The Playful Active Urban Living (PAUL) app is among the first to include 2 types of JIT prompts: JIT adaptive reminder messages to initiate a run or walk and JIT strength exercise prompts during a walk or run (containing location-based instruction videos). This paper reports on the feasibility of the PAUL app and its JIT prompts.

**Objective:**

The main objective of this study was to examine user experience, app engagement, and users’ perceptions and opinions regarding the PAUL app and its JIT prompts and to explore changes in the PA behavior, intrinsic motivation, and the perceived capability of the PA behavior of the participants.

**Methods:**

In total, 2 versions of the closed-beta version of the PAUL app were evaluated: a basic version (Basic PAUL) and a JIT adaptive version (Smart PAUL). Both apps send JIT exercise prompts, but the versions differ in that the Smart PAUL app sends JIT adaptive reminder messages to initiate running or walking behavior, whereas the Basic PAUL app sends reminder messages at randomized times. A total of 23 participants were randomized into 1 of the 2 intervention arms. PA behavior (accelerometer-measured), intrinsic motivation, and the perceived capability of PA behavior were measured before and after the intervention. After the intervention, participants were also asked to complete a questionnaire on user experience, and they were invited for an exit interview to assess user perceptions and opinions of the app in depth.

**Results:**

No differences in PA behavior were observed (*Z*=−1.433; *P*=.08), but intrinsic motivation for running and walking and for performing strength exercises significantly increased (*Z*=−3.342; *P*<.001 and *Z*=−1.821; *P*=.04, respectively). Furthermore, participants increased their perceived capability to perform strength exercises (*Z*=2.231; *P*=.01) but not to walk or run (*Z*=−1.221; *P*=.12). The interviews indicated that the participants were enthusiastic about the strength exercise prompts. These were perceived as personal, fun, and relevant to their health. The reminders were perceived as important initiators for PA, but participants from both app groups explained that the reminder messages were often not sent at times they could exercise. Although the participants were enthusiastic about the functionalities of the app, technical issues resulted in a low user experience.

**Conclusions:**

The preliminary findings suggest that the PAUL apps are promising and innovative interventions for promoting PA. Users perceived the strength exercise prompts as a valuable addition to exercise apps. However, to be a feasible intervention, the app must be more stable.

## Introduction

### Background

Motivating individuals to engage in regular physical activity (PA) is a global interest as physical inactivity can lead to numerous serious health issues such as cardiovascular diseases, cancer, and diabetes [1]. Therefore, individuals are recommended to engage in at least 150 minutes of moderate to vigorous PA (MVPA) every week. In addition, it is recommended to perform bone- and muscle-strengthening exercises at least 2 times a week [2] as they provide additional health benefits next to aerobic exercise [3,4]. However, many individuals do not meet these guidelines [5]. Recent data show that 58.3% of adults (aged >18 years) engage in sufficient moderate PA, and 82.2% engage in sufficient muscle and bone strength exercises, but only 52.9% engage in both [5].

A promising method to increase PA are mobile health (mHealth) PA apps [6,7] such as mobile phone apps. Mobile phones are well integrated into our daily lives [8]; they can continuously track PA behaviors with limited effort from the individual and provide real-time feedback on their behavior. In addition, by continuously tracking the behavior and context of the individual, it is now possible to develop highly personalized and context-based interventions that can offer the right support at the right time [9].

Previous studies have indicated that mHealth PA interventions are more likely to be effective when they are grounded in theory and, as such, contain adequate persuasive strategies [10,11]. Persuasive strategies (or behavior change techniques [12]) are theoretically underpinned elements of interventions intended to facilitate a positive behavior change (eg, rewards and goal setting). It is theorized that persuasive strategies can change the determinants of behavior, such as motivation and capability [13], which in turn influences the targeted behavior. Several studies have demonstrated that self-regulatory persuasive strategies such as goal setting, feedback, monitoring, and prompts are effective in changing PA behavior [7,10,14,15].

In addition, the research fields of human-computer interaction and design thinking emphasize the importance of the quality of the user experience for the success of the intervention [16,17]. *User experience* is an umbrella term that encapsulates concepts such as user satisfaction, perceived usefulness, esthetics, and user-friendliness [18]. Previous studies have demonstrated that apps that have a low subjective user experience are more likely to face low acceptance rates and the problem of nonadherence [19,20]. This is problematic as participants do not engage (sufficiently) with the behavior change components of the intervention [17,21,22] and nonadherence has been shown to negatively influence intervention effectiveness [23,24]. Thus, both the selection of persuasive strategies and their design and implementation are of importance for the success of the intervention [25,26].

A likely effective persuasive strategy is to provide a prompt to engage in a certain behavior [27]. According to the Fogg Behavior Model (FBM) [28,29], the prompt also has to be sent at *the right time* to effectively change behavior. The right time, or *the moment of opportunity*, to send a message is when the individual is motivated enough and when they can perform the exercise. If the motivation and ability are high enough, the individual has crossed the *activation threshold* and can therefore be triggered to perform a behavior. In addition, a well-designed prompt can also increase the motivation (referred to as *spark prompts* [27]) or ability (referred to as *facilitator prompts* [27]) of an individual.

Interventions that aim to send messages at the right time are often referred to as *just-in-time* (JIT) *interventions* or *JIT adaptive interventions* (JITAIs) [9,30,31]. With JIT interventions, the content and timing of the prompt supports the need of the user in real time and is triggered by the system based on predetermined factors. JITAIs are similar except that they also have the ability to adapt the timing or content of the prompt over time to an individual’s changing needs and wishes [9,30,32]. Although prompts are commonly included strategies in PA apps [33], only a few studies have examined the effect of timing on persuasiveness [34,35].

### Objectives

Therefore, we set out to investigate 2 novel ways of JIT prompting for PA behaviors with an mHealth app, the Playful Active Urban Living (PAUL) app. First, to initiate running or walking behavior, the app sends JITAI reminder messages based on a reinforcement learning algorithm [36]. Second, during a PA session (outdoor running or walking), the individual receives JIT location-based strength exercise prompts containing instructional videos for performing strength exercises. These prompts are triggered by either beacons or preprogrammed GPS coordinates, allowing the app to send the right instruction video at the right location and time. For this study, 2 parks in Amsterdam (Sloterpark and Oosterpark) and 1 park in Utrecht (Park Transwijk) were selected as exercise locations. We will refer to these two prompts as *reminder messages* and *strength exercise prompts*, respectively, for the remainder of this paper.

To determine the proof of concept for the design and implementation of the 2 types of prompts, we conducted a feasibility study [37]. Examining the feasibility of a digital intervention before a large-scale effectiveness study is an important step in the development phase. This offers insights into the subjective user experience and engagement with the app and can be used to establish if it is likely that the app will be effective in changing behavior [37,38].

The feasibility of the PAUL app was examined by exploring 4 factors [38]. First, we explored the perceptions and opinions of the users regarding the included persuasive strategies within the PAUL app, with a focus on the 2 novel ways of prompting. Second, the user experience with the PAUL app was examined. Third, we examined the users’ behavioral engagement with the app and, finally, we explored whether the PAUL app has the potential to change the motivation and perceived capabilities of the users. In total, 2 versions of the app were examined: the Basic PAUL app and the Smart PAUL app. Both versions of the app are identical except that the Basic PAUL app sends reminder messages at randomized times, whereas the Smart PAUL app sends JITAI reminder messages based on the context and previous PA behavior of the user.

## Methods

### Participants

Participants were recruited by distributing promotional materials around Sloterpark, Oosterpark, and Park Transwijk. Facebook advertisements were issued targeting individuals aged between 18 and 55 years and living close (<3.5 km) to the parks. In addition, advertisement messages were posted on Facebook resident groups close to the parks (ie, residential groups of apartment buildings). Recruitment materials were also distributed at various universities in the Netherlands and on the social networks of the researchers. The recruitment phase lasted from October 1, 2019, to November 14, 2019.

Initially, we targeted participants aged between 18 and 55 years who lived close (≤1 km; 10-minute walk) to one of the parks used with the beacons (ie, Park Transwijk [Utrecht, Netherlands], Oosterpark [Amsterdam, Netherlands], or Sloterpark [Amsterdam, Netherlands]) and did not meet the PA guidelines of 150 minutes per week (measured using the stages of change questionnaire) [39]. This resulted in too few individuals meeting these criteria; therefore, we changed the eligibility criteria by also including individuals who lived within a 20-minute bicycle ride of the parks (<5 km) and individuals who would like to become more active even if they met the PA guidelines. The exclusion criteria were having a medical condition that made it unsafe to engage in unsupervised PA (defined by the Physical Activity Readiness Questionnaire [40]), not owning an Android smartphone, currently participating in another PA or health-related intervention, or no proficient knowledge of the Dutch language.

### The PAUL Apps

**Figure 1:**
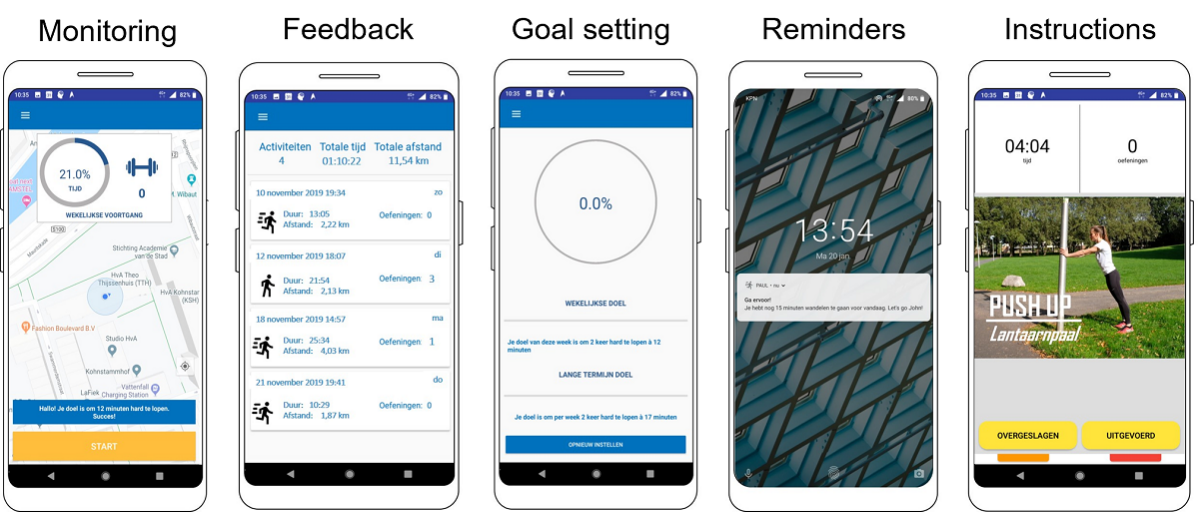
Screenshots of the five functionalities of the Playful Active Urban Living app.

During this study, 2 closed-beta versions of the app were evaluated: Basic PAUL and Smart PAUL. The PAUL apps were developed by a multidisciplinary research team over a 2-year period [41]. The design of PAUL is based on theories of behavior change [13,28], technical implementations and design characteristics [33,42], user studies [43], and data mining studies [44,45]. In short, the PAUL apps are designed to function as a coach to help the user increase recreational walking or running behavior and motivate users to perform additional strength exercises during this walk or run. The apps apply 5 theory-based persuasive strategies: monitoring, feedback, goal setting, reminder messages, and instruction videos (Figure 1).

These persuasive strategies were selected as they are theorized to increase the perceived capability and motivation of the participants based on the Capability, Opportunity, and Motivation Behavior model [13] and the FBM [28]. The theoretical assumptions have been described in detail in an earlier paper on the development of the PAUL apps [41]. Table 1 provides a description of the persuasive strategies that are included in the app, the behavior change techniques [27], and the strategies of the persuasive design model [46]. A detailed description of the app and its development process is provided in the study by Sporrel et al [41] and in Multimedia Appendix 1 [41].

**Table 1:** A description of the modules in the Playful Active Urban Living (PAUL) app, including the implemented behavior change techniques (BCTs) and persuasive system design (PSD) principles.

PAUL functionality, subcategory, and description			BCTs [27]		PSD principles [46]
Strength exercise prompts: The user receives location-based strength exercise prompts (audio and pop-up messages) on predetermined GPS locations. The prompt contains an instruction video of the exercise (squat or push-up) in the direct environment of the user. Amenities in the park (eg, trees, benches, or lantern posts) are used for the exercises.			Information on when and where to perform the behavior Information on how to perform the behavior Demonstrate the behavior Prompt practice		Primary task support: Reduction Tunneling Rehearsal Normative influence
Reminder messages: The user receives up to 14 short reminder messages each week containing a motivational suggestion and either information on the progress toward their goal or (affective) information on performing PA^a^. The timing of the reminder messages depends on the group allocation (Basic vs Smart PAUL).			Information provision (general) Provide feedback on performance Prompt practice		Primary task support: Tunneling Tailoring Personalization Dialogue support: Reminders Suggestions
**Monitoring**					
	PA behavior: The app records and stores PA metrics during app use (frequency, duration, speed, and distance). The user must press “start” to initiate behavior tracking. The app also records and stores situational characteristics during each session and when sending a reminder (weather type, calendar availability, time, and date). After receiving a strength exercise prompt, the user must log if they performed the exercise.	Automatic monitoring of behavior		Primary task support: Reduction Self-monitoring	
	Behavior outcome: The user can report notes on the training session and report on a 1-to-5 scale how they are feeling and how intense the workout was. To monitor how many strength exercises the participant has done, they must log whether they performed or skipped the exercises (during the walking or running activity).	Self-monitoring of behavior Self-monitoring of behavior outcome		Primary task support: Self-monitoring	
**Feedback**					
	Sustained feedback: During running or walking, the user can view simple metrics on their screen (time, distance, current speed, average speed, and number of strength exercises), and the user receives audio feedback every 5 minutes on the duration of the activity.	Provide feedback on performance		Primary task support: Personalization Self-monitoring	
	Cumulative feedback: After performing PA with the app, the user can view a summary of their activities (ie, a PA report) with the time, distance, and average speed and a map with their route. The user can access a history view that contains all PA reports. On the home screen, users can view their progress toward their goal.	Provide feedback on performance		Primary task support: Personalization Self-monitoring	
	Praise: The user receives a pop-up praise message and a message on the landing page when the weekly goal is reached.	Rewards contingent on successful behavior		Dialogue support: Praise	
Goal setting: To set a goal, the user must perform a short questionnaire. With this questionnaire, the user can set their own long-term walking or running goal (for frequency and duration). Furthermore, a tailored start goal (frequency and duration of activity) is given based on the current fitness level of the participant. The goal increases roughly 10% every week until the long-term goal is reached.			Goal setting (behavior) Setting graded tasks Review of behavior goals		Primary task support: Tailoring Dialogue support: Suggestions

^a^PA: physical activity.

The Smart PAUL app differs from the Basic PAUL app in that it can optimize the timing of reminders with a self-learning module [36]. The self-learning algorithm has the opportunity to learn *right* times (ie, JITAI) to send reminders based on the time of the day, the day of the week, previous PA behavior, and agenda availability [36]. Although the timing differed between the Basic and Smart PAUL apps, the content of the reminders was equal.

Both apps were programmed to send up to 14 reminders per week. However, during the intervention period, there were technical issues that prevented the app from sending the reminders (ie, the notifications were not activated in the app because of a processing error in the sent format). For Basic PAUL, this issue was resolved within the first week, whereas, for Smart PAUL, the issue was resolved after 3 weeks. Therefore, the Smart PAUL group only received the JITAI reminders in the last week of the intervention.

### Study Design and Procedures

To determine the feasibility of the PAUL apps, a mixed methods pre-post intervention was performed. This study is part of a larger study that aimed to determine the feasibility of the PAUL apps and examine the user-app interactions with the JITAI reminders. In this paper, we describe the feasibility of the PAUL apps as a whole, whereas we have described the user-app interactions with the JITAI reminder messages in more detail in another paper [47].

Individuals were screened for eligibility using a web-based enrollment questionnaire on the participants’ characteristics. Eligible participants were contacted by the main researcher (KS), and a face-to-face meeting was arranged. During this meeting, the participants were informed about the main objective of the study, the study requirements, and the data handling. When an individual had no further questions, they were asked to sign the informed consent form. The participant then received an accelerometer and an information pamphlet that summarized the most important study information.

All participants started with the baseline measurement either on November 11, 2019, or November 17, 2019. On the day before the start of the baseline measurement, the participants received the baseline questionnaire to assess the determinants of PA and self-reported PA as well as a reminder to wear the accelerometer for 7 consecutive days. A reminder to fill in the web-based questionnaire was sent when needed after 2 days.

After successful completion of the baseline period, the participants were manually randomized into the Smart and Basic PAUL groups by an independent researcher with a 1:1 ratio stratified by the 3 parks (ie, Park Transwijk, Sloterpark, and Oosterpark). The group allocation was double-blinded. In the following 4 weeks, the participants could use the PAUL app. The user was asked but not obligated to not turn off the reminder function (ie, push notifications) of the app and to give access to their digital calendar. The participants were informed that this would improve the function of the app without explaining any details of the differences between the 2 groups. During the intervention, the participant received a visit from a researcher to download the accelerometer data.

At the start of the fourth intervention week, the user was reminded to wear the accelerometer again for 1 week. After 5 weeks, the individual received a link to the final questionnaire on the usability of the PAUL app, the determinants of PA, and the self-reported PA. After the intervention, all the participants were invited for an interview at a location of their liking. As a token of appreciation for participating in this study, the participants received a voucher for a cinema visit or a sports activity with a value of €30 (US $31.32).

### Measurements

#### Perceptions and Opinions of the PAUL App

To gain a better understanding of the users’ perception of the PAUL app and its functionalities, all participants were invited for semistructured exit interviews of 20 to 40 minutes at a location of the participants’ choice. The topics in the interview guide covered the perceptions of the included strategies, the design and implementation of the strategies, and the user experience of the strategies. During the interviews, the researchers and participants were still blinded to their group allocation.

#### User Experience

In addition, a web-based, 20-item, 7-point scale questionnaire on the user experience with the PAUL app was administered at the end of the intervention (acquired from the study by Mollee et al [18]). The questionnaire contained 4 subtopics—perceived effectiveness, usability, satisfaction, and esthetics. The participants could state how much they agreed with the subtopics, from 1 (not at all) to 7 (completely). An item was added to the questionnaire on how many technical problems they encountered.

#### Behavioral Engagement With the PAUL App

To determine how often the participants used the app during the intervention and, thus, were exposed to the intervention strategies (referred to as *behavioral engagement* [17] or *sustained use* [48]), we examined the number of times the users opened the apps on each intervention day. This was registered automatically by the apps.

#### Intrinsic Motivation and Perceived Capability

Perceived capability and intrinsic motivation were measured independently for the 3 behaviors of the app (running, walking, and strength exercise). The 6-item perceived competence subscale of the Intrinsic Motivation Inventory [49] was used to measure capability, and the 7-item interest and enjoyment subscale was used to measure intrinsic motivation. The subscales of the Intrinsic Motivation Inventory were back translated into Dutch. Each item could be scored from 1 to 7, with a score of 1 indicating *not at all true* and a score of 7 indicating *very true*. Subscale scores were calculated by reversing 3 items and subsequently averaging the items of the subscales.

#### PA Measurement

The PA behavior of the participants was measured using a hip-worn accelerometer, the ActiGraph GT3X+ (ActiGraph LLC), 1 week before the intervention (baseline) and in the last week of the intervention (after the intervention). Accelerometer measurements were considered sufficient if the participants wore the accelerometers for a minimum of 8 hours a day and for at least 3 weekdays and 1 weekend day. A total of 35% (8/23) of the participants did not meet these requirements for either the pre- or posttest measurement and were therefore excluded from the analysis.

### Analysis

#### Perception of the PAUL App

The interviews were audio-recorded on the researchers’ phones and transcribed verbatim. After transcribing the interviews, the text was imported into MAXQDA Plus (version 20.2.2; VERBI GmbH). Qualitative research cycle was used to code and analyze the data [50]. Before the analysis, a first version of the codebook was developed based on the topic list. Therefore, most codes were developed deductively. The transcripts were then coded. When new topics emerged from the interviews, they were added to the codebook. To ensure that the same coding system was used for all interviews, they were coded again after new codes were derived from the data. Memos were used and served as reminders to explore links between certain codes or to compare conflicting statements.

After coding all the data, the codes were analyzed by a researcher according to a cyclic process [50]. This included regrouping the codes into larger categories (such as *general perceptions of reminders* or *personal support*), exploring links between codes and categories (such as *weather constraints*, *goal setting*, and *reminders*), and comparing between participants. Codes were sometimes also uploaded to Microsoft Excel and subdivided into smaller categories (eg, positive and negative perceptions). The findings for each of the codes and categories were summarized. As this was a cyclic process, the codes were regrouped several times, sometimes into larger categories, and sometimes the codes were divided into subcodes.

#### User Experience

In addition to the interview data, the questionnaire responses were used to gain an overall perspective on the technical problems, perceived effectiveness, usability, satisfaction, and esthetics of the app. To this end, the questionnaire responses were uploaded to SPSS (version 25; IBM Corporation), and the item on technical issues was inverted. To determine the differences between the Smart and Basic PAUL apps, a Mann-Whitney *U* test was performed (1-tailed exact test with significance at *P*<.05). A participant did not complete this questionnaire and was therefore not included in the analysis.

#### Behavioral Engagement With the PAUL App

The behavioral engagement with the PAUL app data was uploaded from the servers of the PAUL app and subsequently cleaned by removing duplicate data. The data were validated by cross-checking the data sets of the PAUL app. For some participants (3/20, 15%), no data were recorded by the app. This was likely due to a connection error with the back end of the app, which could be caused by several factors such as battery failure. Participants whose data were not recorded could not be included in the analysis. Descriptive statistics were calculated for the participants in the Basic and Smart conditions. Differences between the Smart and Basic PAUL apps were calculated using a Mann-Whitney *U* test with an exact test (1-tailed significance at *P*<.05).

#### Intrinsic Motivation and Perceived Capability

To analyze the changes in intrinsic motivation and perceived capability, we only included the measures for behavior that the participants wanted to change. That is, if the participants had set a goal with the PAUL app to increase their walking activity, we only used their scores for walking. If they had a running and walking goal, the scores were averaged. This also included changes that the participants made during the intervention. The scores for motivation and perceived capability to perform strength exercises were calculated for all participants. The scores were then uploaded to SPSS, and a descriptive analysis was performed. To determine if there were differences between the groups, the differential scores between the pre- and postintervention measurements were calculated, and Mann-Whitney *U* tests were performed (1-tailed exact test with significance at *P*<.05). To determine differences between pre- and postintervention measurements, Wilcoxon signed-rank tests were performed (1-tailed exact test with significance at *P*<.05). Nonparametric tests were used because of kurtosis in the data.

#### PA Analysis

To process the accelerometer data, they were downloaded using ActiLife (version 6.13.4, firmware 2.2.1; ActiGraph LLC), and the triaxial counts were summed as counts per minute (cpm). Episodes of at least 90 minutes were defined as nonwear episodes. Short interruption periods of a maximum of 2 minutes of 1 to 100 cpm were allowed as nonwear time to account for the possibility of accidental accelerometer movement. Only days with at least 8 hours of wear time were included in the analysis. Freedson Adult (1998) cutoff sets were used to define the time that the participants spent on MVPA (>1951 cpm). The average MVPA time was calculated while excluding nonwear time. To account for the differences in wearing time, the average time spent performing MVPA was calculated. The PA measurements were imported into SPSS, and a Mann-Whitney *U* test with the differential scores was used to determine the differences between Smart and Basic PAUL. A Wilcoxon signed-rank test was used to establish differences between pre- and postintervention measurements (both 1-tailed exact tests with significance set at *P*<.05).

### Ethics Approval

The study method was approved by the local ethical committee (GEO S-19253), and the trial was registered in the Netherlands Trial Register (trial ID: NL8166). The study was conducted and reported according to the CONSORT-eHEALTH (Consolidated Standards of Reporting Trials of Electronic and Mobile Health Applications and Online Telehealth) checklist [51].

## Results

### Participants

**Figure 2:**
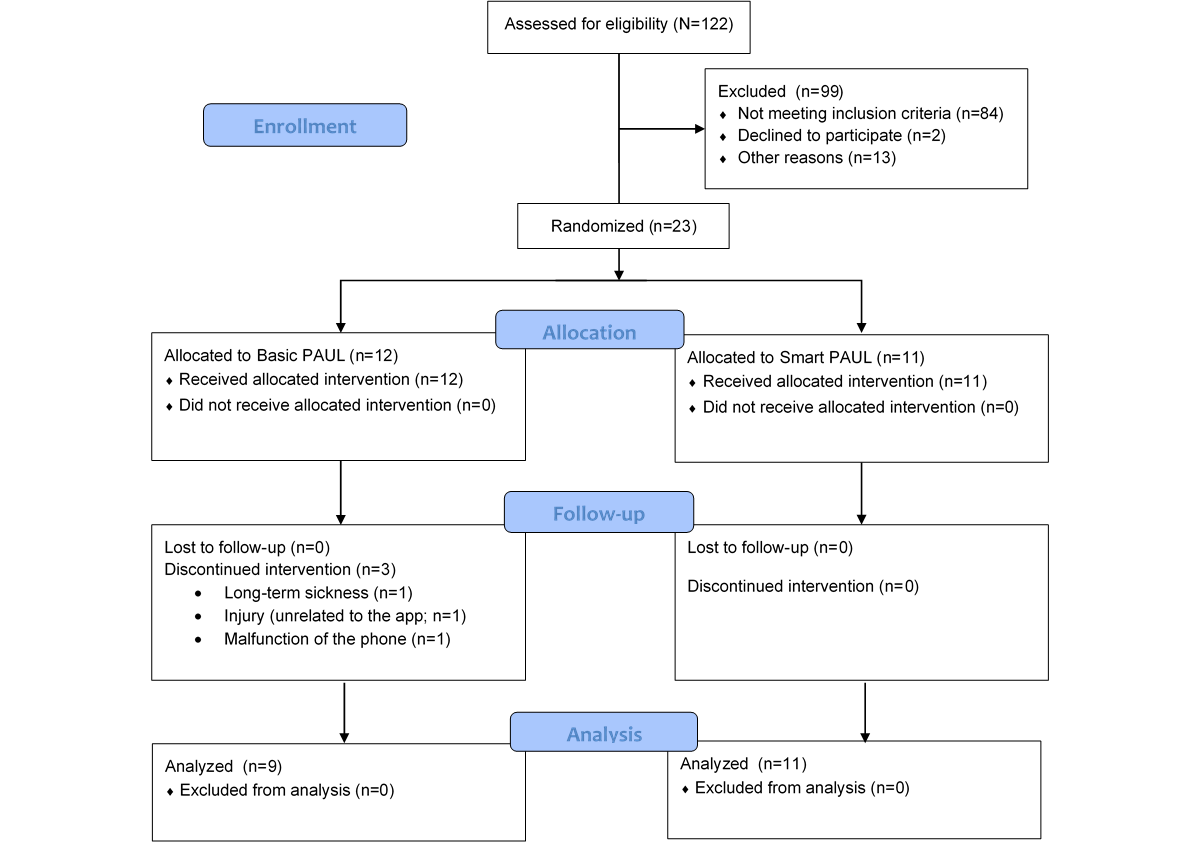
CONSORT (Consolidated Standards of Reporting Trials) flow diagram of participants. PAUL: Playful Active Urban Living.

Recruitment resulted in 122 individuals who were interested in participating in the study and completed the enrollment questionnaire. After checking eligibility and provision of informed consent, of the 122 interested individuals, 23 (18.9%) were enrolled in the study. The main reasons for exclusion were that the individuals did not live close enough to the parks equipped with beacons or did not own an Android phone. Of the 23 participants, 3 (13%) discontinued their participation, leaving a total of 20 (87%) participants who completed the study. The participant flow diagram is shown in Figure 2.

The characteristics of the included 20 participants are shown in Table 2. Most participants were women (17/20, 85%), had an average age of 30.65 (SD 8.4) years and an average BMI of 24.52 (SD 5.23) kg/m^2^, and were highly educated (16/20, 80%) and employed (14/20, 70%). Many participants engaged in regular moderate PAs (14/20, 70%), but most participants did not engage in intensive PAs (15/20, 75%).

**Table 2:** Background characteristics of the participants (N=20).

		All (N=20)	Smart PAUL^a^ (n=11)	Basic PAUL (n=9)
Gender (female), n (%)		17 (85)	9 (82)	8 (89)
Age (years), mean (SD)		30.65 (8.40)	32.09 (10.73)	28.89 (4.17)
BMI^b^ (kg/m^2^), mean (SD)		24.52 (5.23)	25.79 (6.49)	22.79 (2.04)
**Education level, n (%)**				
	Secondary school (VWO^c^)	3 (15)	2 (18)	1 (11)
	Vocational education	1 (5)	1 (9)	0 (0)
	Higher professional education degree	3 (15)	2 (18)	1 (11)
	University degree	13 (65)	6 (55)	6 (67)
**Housing, n (%)**				
	Living alone	8 (40)	3 (27)	5 (56)
	Living alone with children and others	1 (5)	1 (9)	0 (0)
	Living with partner	3 (15)	0 (0)	3 (33)
	Living with partner and children	3 (15)	2 (18)	1 (11)
	Living with partner, children, and others	1 (5)	1 (9)	0 (0)
	Living with more adults (such as student housing)	4 (20)	4 (36)	0 (0)
**Employment status** **,** **n (%)**				
	Part-time employment (<34 hours per week)	8 (40)	4 (36)	4 (44)
	Full-time employment (≥34 hours per week)	6 (30)	3 (27)	3 (33)
	Studying	6 (30)	4 (36)	2 (22)
**Stage of change (moderate PA^d^), n (%)**				
	Maintenance phase	13 (65)	6 (55)	7 (78)
	Action phase	1 (5)	0 (0)	1 (11)
	Preparation	2 (10)	2 (18)	0 (0)
	Contemplation	4 (20)	3 (27)	1 (11)
**Stage of change (strength exercises), n (%)**				
	Maintenance phase	5 (25)	3 (27)	2 (22)
	Action phase	0 (0)	0 (0)	0 (0)
	Preparation	5 (25)	3 (27)	2 (22)
	Contemplation	8 (40)	4 (36)	4 (44)
	Precontemplation	2 (10)	1 (9)	1 (11)
**Running experience, n (%)**				
	No or little running experience	5 (25)	3 (27)	2 (22)
	Experienced runner, not currently running	12 (60)	6 (55)	6 (67)
	Experienced runner, currently running	3 (15)	2 (18)	1 (11)

^a^PAUL: Playful Active Urban Living.

^b^The weight of 1 participant was entered incorrectly and was therefore not included in this table.

^c^VWO: Voorbereidend wetenschappelijk onderwijs (preuniversity education).

^d^PA: physical activity.

### PA Behavior and Determinants of PA Behavior

**Figure 3:**
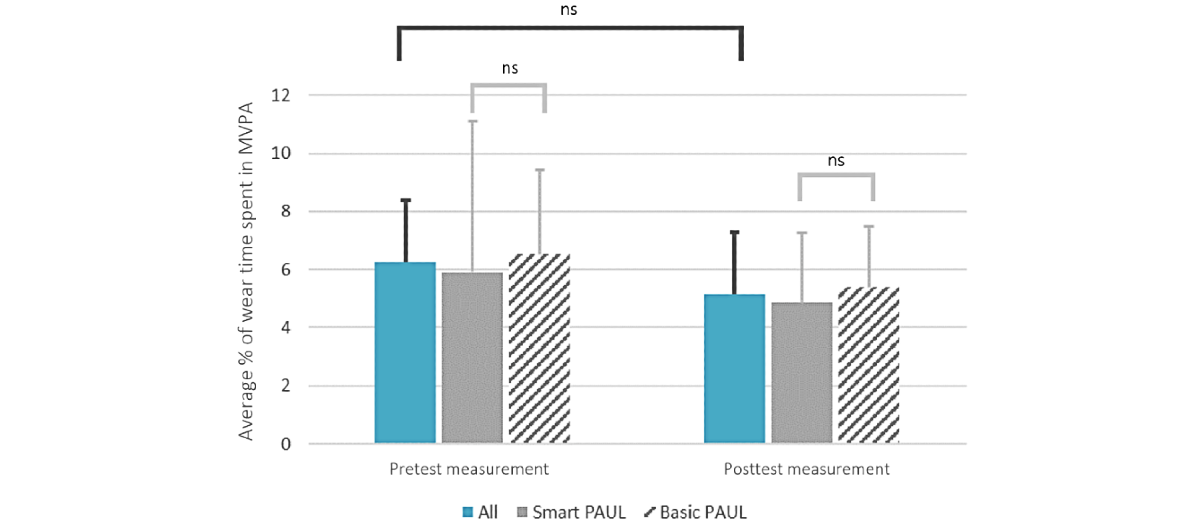
The pre- and postintervention MVPA time for the Smart and Basic groups. MVPA: moderate to vigorous physical activity; ns: not significant; PAUL: Playful Active Urban Living.

To examine to what extent the PA behavior of the participants changed over time, the accelerometer data of the participants were analyzed. A total of 35% (7/20) of the participants were excluded from the analysis as they did not meet the required wear time. For the remaining 65% (13/20) of the participants, there were no significant differences between the participants in the Smart PAUL group and the Basic PAUL group (*U*=16.00; *z*=−0.714; *P*=.53) in the differential scores of MVPA time (Figure 3). As there were no differences between the Smart and Basic PAUL apps, the 2 groups were treated as 1 to determine the differences in PA pre- and postintervention measurements. As shown in Figure 3, the percentage of time spent in MVPA slightly decreased over time, but no significant differences were found (*Z*=−1.433; *P*=.08).

**Figure 4:**
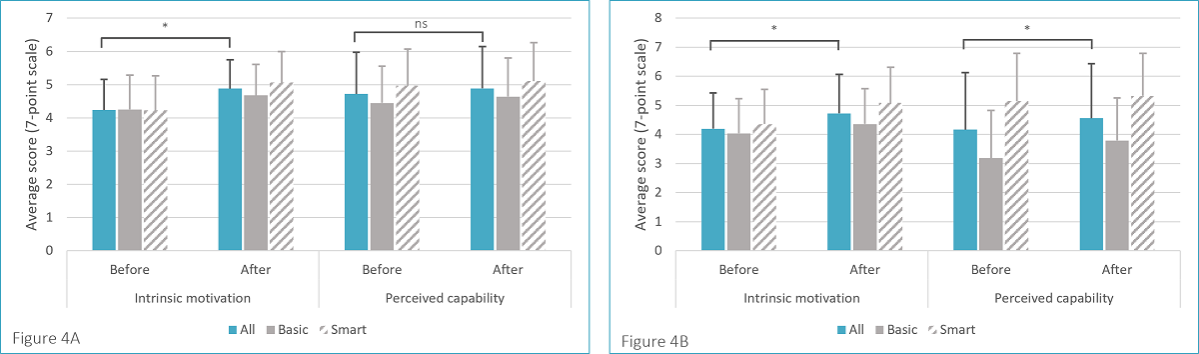
The pre- and postintervention intrinsic motivation (4A) and perceived capability scores (4B) of the participants enrolled in the Basic and Smart Playful Active Urban Living groups. ns: not significant.

Next, intrinsic motivation and perceived capability were examined for running or walking and strength exercises (Figure 4A and Figure 4B, respectively). The Mann-Whitney *U* test did not show differences between the groups in running and walking behavior motivation (*U*=36.000; *z*=−0.736; *P*=.24) and capability (*U*=41.000; *z*=−0.327; *P*=.38) or in strength exercise motivation (*U*=30.500; *z*=0.158; *P*=.45) and capability (*U*=18.000; *z*=−1.474; *P*=.08). Thus, the Smart PAUL and Basic PAUL apps appear to influence motivation and capability equally.

To determine the differences in determinants before and after the intervention, a Wilcoxon signed-rank test was performed. As there were no differences between the 2 groups, we analyzed the 2 user groups as 1. Significant differences were found in running and walking motivation (*Z*=−3.342; *P*<.001) but not in capability (*Z*=−1.221; *P*=.12). Regarding the performance of strength exercises, both motivation (*Z*=−1.821; *P*=.04) and capability (*Z*=2.231; *P*=.01) significantly increased.

### User Experience

**Figure 5:**
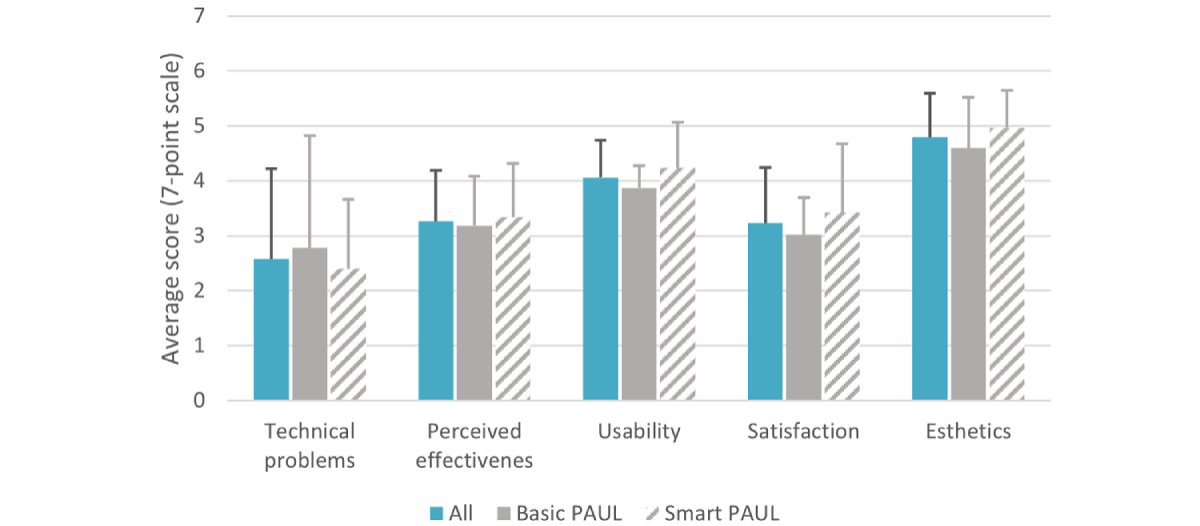
User experience ranging from 1 (low) to 7 (high) of the Smart and Basic PAUL apps. PAUL: Playful Active Urban Living.

The user experience was examined using a questionnaire in which the user was asked to rate the app on a score from 1 (lowest) to 7 (highest). As shown in Figure 5, there were no differences in user experience between the 2 groups in terms of technical problems (*U*=41.50; *z*=−0.298; *P*=.41), perceived effectiveness (*U*=40.5; *z*=−0.369; *P*=.37), usability (*U*=27.0; *z*=−1.485; *P*=.07), satisfaction (*U*=31.0; *z*=−1.15; *P*=.13), and esthetics (*U*=33.5; *z*=−0.948; *P*=.18). Both groups reported that they experienced many technical issues (mean 2.60, SD 1.64), which likely also resulted in a low perceived effectiveness of the app in changing their PA behavior (mean 3.26, SD 0.91) and a low satisfaction with the app (mean 3.23, SD 1.01). Compared with perceived effectiveness and satisfaction, the participants were more positive in terms of the usability (mean 4.06, SD 0.67) and esthetic appeal of the app (mean 4.79, SD 0.80).

### Behavioral Engagement With the PAUL App

**Figure 6:**
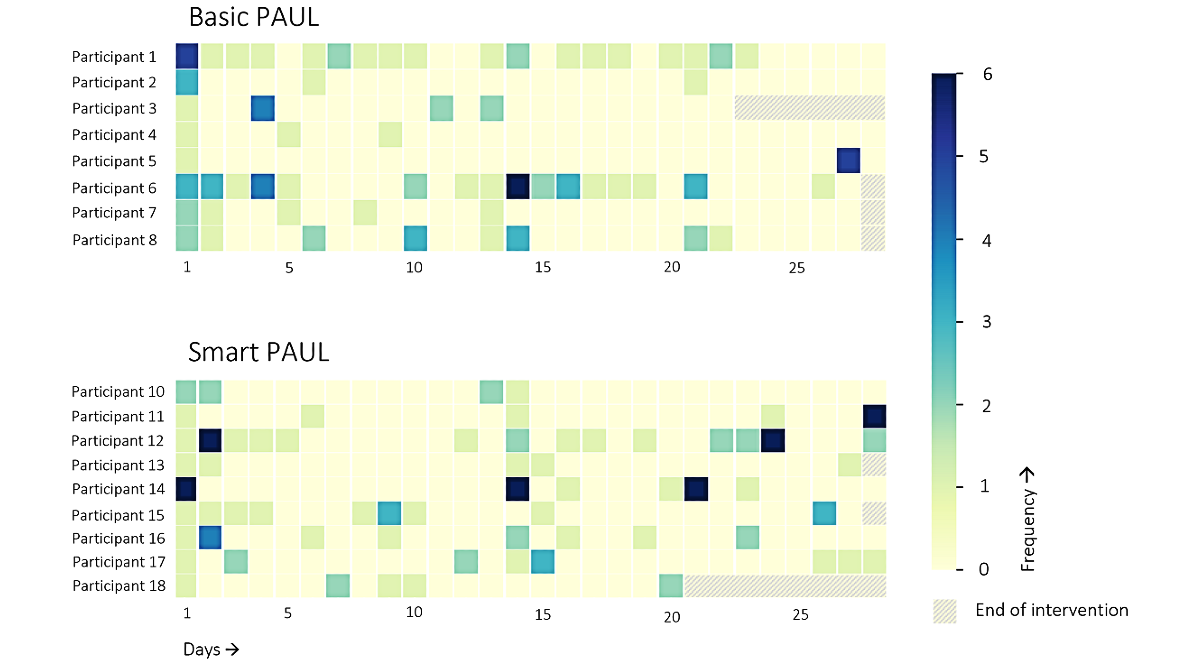
The daily frequency of opening the PAUL app from the day the Basic and Smart PAUL apps were downloaded. PAUL: Playful Active Urban Living.

To examine whether the participants used the app and, thus, were exposed to the included persuasive strategies, we explored the frequency of opening the app. As can be seen in Figure 6, there were a few frequent users of the PAUL app, and most participants opened it a couple of times a week. On average, the participants opened the app on 7.3 (SD 4.67) days of the 28 intervention days, with the most frequent user opening the app on 19 days and the least frequent user opening it on 2 days. There were no significant differences between the 2 groups regarding the total frequency of opening the app (*U*=28.5; *z*=−0.723; *P*=.48) or the number of days the apps were open (*U*=27.0; *z*=−0.875; *P*=.42). However, it does seem that the Smart group continued using the app for a longer period as opposed to the Basic group. A likely explanation is that the Smart group started to receive the reminders in the last week, whereas the Basic group received the reminders throughout the intervention.

### Perceptions and Opinions of the PAUL Apps and the JIT Prompts

#### Overview

In this section, we describe the findings of the exit interviews. The perceptions and experiences of both PAUL apps are reported simultaneously to a large extent as the app functionalities are also largely similar. Only the results regarding the timing of the reminders are reported separately.

#### Perceptions and Opinions of the PAUL Apps

Overall, the participants thought that the included persuasive strategies were useful and that the most important features were present in the app. The app was perceived by most as “simple” and “basic,” which was liked by some of the participants as it made using the app clear with a simple goal:

And furthermore, it’s nice that it’s so clear, that you’re not lost, or that you’re somewhere in 7 steps and think: where am I? And that was nice. He was just clear.

Participant 6, female, aged 35 years

Other participants indicated that they preferred a more elaborate app:

Um, all right in the general sense. But, um, in many ways, just a little basic. In terms of what you can do with it, of course.... I went running with my girlfriend during this research and she has a Nike running app, and yes, that's super interactive.

Participant 18, female, aged 22 years

Well, I thought it was, in its essence, a really nice app to use, for instance for running. Here and there, there were some features of which I thought: “Oh those should be further developed.”

Participant 5, male, aged 32 years

The improvements to the app mainly lay in the development of the implementation of a strategy rather than the addition of another strategy (eg, earning coins or a leaderboard). For instance, participant 18 (female, aged 22 years) would have liked more “interactions” with the app (eg, controlling whether she could perform the strength exercises in a particular exercise session and how many she could perform). By doing so, the users can personalize the apps themselves. Furthermore, the improvements to features that participant 5 (male, aged 32 years) mentioned were to provide more detailed and graphic information about his activities and add more types and locations for the strength exercises. Other participants would have liked “to know the idea behind” the goal setting functionality (participant 8, female, aged 30 years); thus, she would have liked additional information on how the goal was determined.

A frequently mentioned improvement in functionality was to automatically track all the PA activities of the user. Some participants hoped for an app that helped them integrate more PA into their daily activities (eg, cycling slightly further than normal or taking the stairs more often) as this fit better in their life than going for a recreational run or walk. Furthermore, as not all their activities were recorded, they felt as though the app did not give them credit for all their PA behavior:

What I also find unfortunate about it was that you physically had to say that you’re going to move now. And a lot of my movements just happen in life, so to speak. So, when I walk to the supermarket, or when I go there or there for a bit. And I’m not gonna enter that. And he won’t record that. Whereas for me, those are the moments that I could make a profit, that if he would record it.

Participant 6, female, aged 35 years

In addition, we asked about the perceptions of 3 frequently used persuasive strategies in apps that were not included in the PAUL apps: rewards (eg, victory points, digital coins, or digital awards), social support, and competition. A few participants indicated that they had nothing against rewards but that they also did not see their added value. More than half of the participants indicated that they would like to be rewarded, but they explained that receiving feedback on their progress or on the number of activities they had done was already enough of a reward. The participants expected that such rewards would strengthen the feeling of having a competition with themselves. For some participants, this was perceived as motivating, whereas others were afraid to disappoint themselves.

Competition with others was disliked by most participants. A total of 10% (2/20) indicated that they would like competition with their friends as this was a fairer comparison and, therefore, more achievable. Sharing exercise outcomes on social media was disliked as this was viewed as a call for attention. A participant indicated that sharing running routes (within the app) would be a good addition to the app. Some participants also suggested other functionalities by themselves. These were to have a selection of running routes, information on why it is important to engage in PA, emails regarding progress, and a game element to motivate users to visit certain exercise locations.

Although the participants in general explained that the app contained the most important strategies, most participants reported that they only used the app a few times. Mostly, they stopped using the app because they encountered technical issues. There were also participants who explained “that’s not the apps fault” (participant 6, female, aged 35 years) that they stopped using the app as they encountered barriers such as lack of time or bad weather. For instance, various participants explained that, because of the short winter days, it was already dark when they got home from work. This, together with colder and wetter weather circumstances, made it unpleasant to go outside for a run or walk:

Only I have to say that I used it less than I had hoped, because it was often bad weather, and very dark. And then you’re less inclined to go outside. Normally earlier in the summer I would do something more quickly anyway.

Participant 19, female, aged 28 years

When asking the participants how an app could help them overcome these barriers, they found it difficult to give an answer. After debating the issue, some suggested receiving encouragement to go for walks when there was still daylight; for instance, during lunch. Another participant suggested including a module that enabled them to perform the strength exercises simply at home in case they were bound to stay there to watch their children or when the weather was bad.

Although some barriers to the uptake of the app lay outside the app, the biggest issue with using it were the technical problems the participants experienced. When encountering a technical problem, this evoked *irritation* and *frustration* in the users or the feeling that their efforts were not recognized by the app or of not being encouraged enough to do more activities. This ultimately resulted in app abandonment. For some participants, it even resulted in thinking that they had done something wrong (participant 5, male, aged 32 years). Various participants explained that, if the app had been more stable, they would have probably used it more often.

#### Perceptions and Opinions Regarding the Strength Exercise Prompts

Many participants did try out the location-based strength exercises. Of all the strategies that were implemented in the app, the participants were generally the most enthusiastic about the strength exercise prompts:

Well, the best part was that when I just walked through the park, that I always got one, one.... What’s that called? That I got a sound [strength exercise prompt], and then I had to do something. I really liked that about it.

Participant 17, male, aged 25 years

Owing to the novelty of this functionality, the participants became curious and motivated to try it out. Some participants were motivated to perform these strength exercises to increase their strength and fitness. According to these participants, complementing their running activity with strength exercises resulted in a more complete workout in which they trained not only their endurance but also their strength. Furthermore, receiving a strength exercise prompt was perceived as receiving a surprise, some kind of *game* or reward for going outside for a run or walk. The participants explained that it made a running or walking session more diverse and, therefore, more fun. Some participants even referred to the strength exercises as “a moment to catch your breath” for running. In addition, the participants enjoyed that the app gave suggestions on what they could do, similar to a coach, so they did not have to figure everything out themselves:

But I liked the mix, and that it’s bound to certain parts of the park, so to speak. That also, when you do an exercise, it can tell you where to do it. So, yeah, I actually really liked that.

Participant 5, male, aged 32 years

The app could give better suggestions for the strength exercises as it “knew” where the participant was. Some participants explained that it helped them view the park amenities (eg, benches) in a different light, as something they could use during their workout:

Well, what I found very interesting was the part of, uhm, location. That it would indicate those strength exercises at the right places, so to speak.

Participant 8, female, aged 30 years

To *know* where the user is, the user must share their privacy-sensitive location data with the app. Although most participants did not express any concerns regarding privacy, others explicitly said they preferred not to do this. Several reasons were brought up, including mistrust of who manages these privacy-sensitive data. For instance, some participants were very hesitant to share data with commercial companies, whereas they were willing to share the data with the government or universities:

Well, if it’s not commercial, then maybe I would. It depends. Where’s it all going, huh? What does that app need to analyze it all?

Participant 16, female, aged 42 years

In addition, some participants also considered that the privacy-sensitive data that are collected by the app must be of added value to them. In other words, they were willing to share data if they obtained something they wanted from them in return. For instance, one of the participants explained what he liked about the strength exercise prompts:

So, I guess that it shows you: you’re here now, so it shows you the [strength exercise] possibilities. Somewhere it’s a bit freaky, that he can follow me anywhere, but assuming that privacy is well guaranteed, it delivers a lot.

Participant 10, male, aged 28 years

Although the app removed some barriers, other barriers remained. Some female participants mentioned that they would not perform the strength exercises as they did not like to do them in such public spaces were other people could “look at you like that” (participant 1, female, aged 27 years and participant 3, female, aged 23 years). Other participants expressed their concern about performing strength exercises without receiving feedback on their posture from a professional. Offered solutions to this problem were to organize a group training, only implement easy exercises, or motivate the participants to practice the exercises at home in front of a mirror. Another improvement that was suggested was offering the strength exercises in more locations so the participants did not have to travel to the location before they could start their run or walk. The participants would also like to have more types of strength exercises and combinations of strength exercises that could be tailored to their capabilities. Some participants indicated that they would like to see where the strength exercises were located so they were motivated to run there, “explore” the neighborhood, and discover new exercise locations.

#### Perceptions and Opinions Regarding the Reminder Messages

Reminder messages were perceived as important initiators of behavior by almost all participants. The participants said that the reminders “trigger” them (participant 20, female, aged 23 years) and that it “lowers the threshold” to engage in PA (participant 5, male, aged 32 years) and, therefore, increases the chances of engaging in PA. Thus, to some extent, the reminders function as a coach (ie, something that pulls the participants over when they have difficulty in performing the behavior themselves). Unfortunately, not all the participants received the reminder messages. However, these participants also explained that they thought they would have used the app more if they had received them. As they did not receive the reminder messages, they often forgot the app and their intention to exercise more.

Some participants explained that a reminder message in itself was not enough to motivate PA. Rather, the motivation must come from within, and the reminder message can help overcome barriers or remind them of their plans:

Yes, then it’s nice that one of those things reminds you of it, but then you think “yes, but I just don’t have time for it right now”.... So I’m more like, “yes, I’d like to try,” but it doesn’t really fit in my schedule. So, then it should be a bigger mind set of “okay, I think this is very important,” that you really make that app a part of your daily rhythm, yes.

Participant 10, male, aged 28 years

The participants highlighted the need for well-timed and highly personalized reminder messages. Although receiving a reminder message at a good time could serve as a trigger, receiving one at a bad time could lead to irritation or feeling like they were failing (participant 20, female, aged 23 years and participant 17, male, aged 25 years). The timing of the reminder message also seemed to influence the perception of its content. For instance, a participant explained that the reminder message was annoying and “preachy.” Owing to a busy schedule, she did not have the time to go for a walk even though she had the motivation to exercise. Thus, a motivating message was not appropriate. However, she explained that she would design similar reminder messages herself as, if they were well timed, they entertained her. Furthermore, some participants explained that the content of the reminder message did not really matter at all and that receiving a prompt in itself was already sufficient. In line with these comments, several participants had issues with recalling the content of the reminder messages, indicating that the content indeed was not the most important quality of the reminder.

As the timing of the reminder message was perceived as important, the participants enjoyed the idea that the app knew their schedule by reading their agenda:

I think that’s a plus compared to other things. That you can then, that you can link it that way.

Participant 8, female, aged 30 years

However, some participants did express privacy concerns or did not use a digital agenda that could be integrated into the app.

During the interviews, participants in both groups were generally not positive about the timing of the reminder messages. The perceptions of the timing of the JITAI reminder messages were not more positive than those of the randomly timed reminder messages. A possible explanation for these findings is that the participants in the Smart PAUL group received too few reminder messages as they only received them in the last week of the intervention. Furthermore, owing to the short study duration, the reinforcement learning model could only use a prelearned delivery strategy to determine the timing of the messages. Thus, it was not able to adjust the strategy at an individual level.

As the participants were not satisfied with the timing, we discussed what they would have preferred. It seems that there were roughly 2 groups of participants—one group that liked to set their own times and one group that wanted to receive regular reminder messages throughout the day:

...if I already know that I can’t run that day at all, because I must do all kinds of other things, then I think it would only be annoying that I would still get reminders for something.... But I am also someone who then, plans in advance which days she wants to walk, so to speak.

Participant 16, female, aged 42 years

I’m not a planner, so, um, I get a bit itchy when I’m very tightly planned and know what I’m gonna do on what day. Especially when it’s in my spare time. Um, so, I’d rather get [a reminder] every day. That sometimes you think: “oh, yeah, I want to work out.” And sometimes I don’t.

Participant 18, female, aged 22 years

Participants who claimed that they always planned their (physical) activities liked to set times at which they wanted to receive the reminder message. In contrast, participants with a more flexible agenda or who did not like to plan liked to receive regular and well-timed reminder messages throughout the day and decide on the spot whether they wanted to exercise.

## Discussion

### Principal Findings

The feasibility of the Smart PAUL and Basic PAUL apps was examined by exploring the users’ perceptions of the app, experiences, behavioral engagement and changes in PA behavior, and determinants of PA behavior. The main findings of the study were that the participants appreciated the included persuasive strategies, especially the strength exercise prompts. The strength exercises were motivating because of their novelty and because they offered variety during a run or walk and a more complete workout. Some participants even perceived the strength exercise prompt as a reward. Furthermore, the reminder messages were perceived as important initiators for PA by most participants, but they were not perceived as well timed.

Another finding was that there were little to no differences between the Smart PAUL and Basic PAUL groups regarding perceptions, opinions, and user experience. This is likely the result of the small difference between the 2 versions of the app, which became even smaller as the reminder messages were not sent during the first part of the study. Owing to this short duration, the Smart PAUL app could only apply a prelearned strategy to determine the timing of the reminders and was not able to adjust the timing to each individual participant. Finally, we found no improvement in the PA behavior of the participants, but we did find an increase in the perceived capability to perform strength exercises, and the intrinsic motivation for walking, running, and performing strength exercises did increase during the intervention. Taken together, we conclude that the PAUL apps are not feasible interventions in their closed-beta state but, if they were more stable, they could be effective in promoting PA.

The increased motivation for running, walking, and performing strength exercises and capability to perform strength exercises supports the theoretical assumptions on which the PAUL apps were based [41]. However, despite the improvement in the targeted constructs, no increase in the PA behavior of the users was observed. A possible explanation is that PA behavior will only increase if other determinants, such as opportunity [41], increase as well. Another possibility is that the motivation or capability of the user must increase even more to influence PA behavior. Notably, these results must be interpreted with caution because of the small sample size and the lack of a control group. For instance, it is possible that, without the app, PA behavior would have decreased. A randomized controlled trial with a no-treatment control group should be performed to examine this.

By examining the feasibility of the PAUL app, we could explore the perceptions of 2 different types of JIT prompts: one to *initiate* the exercise session and one to initiate a specific behavior *during* exercise. The participants appeared to be especially receptive to the strength exercise prompts during exercise. A likely explanation for this is that, because the participants were exercising, they were already motivated, whereas the prompt itself increased the (perceived) capability of the participant to perform the exercise (ie, it is a facilitating prompt [27]). As explained by the participants, the prompt ensured that they did not have to think about what they should do. At the same time, the prompt nudged the participants to perform the behavior. Consequently, the strength exercise prompt could push the participants over the *activation threshold* while also triggering the behavior [28,29].

By including prompts to initiate a behavior during exercise, the PAUL app is among the first to make a combination of strength exercises and aerobic exercises. Thus far, only 2 other apps have been developed and evaluated: MOPET [52,53] and eCoFit [54,55]. Both MOPET and eCoFit offer exercises at fixed locations to which the participant must travel to exercise. However, based on the interviews in this study, having to travel to a different location could be a barrier to performing PA. Furthermore, the participants expressed a need for variation in terms of going to different locations and choosing different routes to run or walk and different strength exercise types. Thus, to keep an app interesting and surprising, future apps should enable users to use it everywhere. Notably, little is known about which outdoor locations could support the performance of strength exercises and which exercises are suitable. Therefore, future research is needed to examine this to optimize and improve this functionality.

The participants appeared to be less receptive to the second type of prompts, the reminder messages. The reminder messages were often sent when the participants did not have the opportunity or capability to exercise even though they were motivated to do so. Moreover, as the motivation message only aimed to motivate the participants and not to increase their capability, it was not *the right* message at *the right time*.

The interviews suggested several options to improve the reminder messages that support the FBM [29]. First, to increase the (perceived) capability to exercise, for instance, the exercises can be made easier to achieve by making it possible to do them at home. Second, the timing could be better tailored to the moments of opportunity of the individual (eg, when they do not have other obligations such as childcare or work). To this end, a future system should at least include calendar availability, weather, daylight, amount of PA performed, and the users’ (exercise) routines according to the participants. Calendar availability, weather, and the amount of PA performed have been used in earlier studies on JIT reminders [34,56,57], but daylight and routine, to the best of our knowledge, have not been used. In addition, future systems should have longer periods to collect more data to learn the optimal strategy.

The need for a smart and personalized system contrasts with the need for privacy. The participants were very clear on why it is important to have state-of-the-art technology—to provide very personal support that contains the exact types of support they need. This recognizes that every individual is different and has different needs. However, some participants were hesitant to share the data that are needed for this type of support. In line with earlier research [58,59], participants in this study also made a careful trade-off in which the benefits must outweigh the costs (referred to as the *privacy-personalization paradox* [59,60]). Costs that were too high for some participants were the feeling that their information could be misused or that people could make a profit from their data. The participants explained that they would be willing to share data if they had a great added value in their lives or when their curiosity overruled the costs. Thus, careful considerations must be made when working with such technologies in terms of the information that is needed and the potential consequences, such as the exclusion of a part of the target group.

### Strengths and Limitations

There are some limitations to this study. A major limitation of this study were the technical issues that overshadowed the results of the feasibility trial. This demonstrates that the beta version of the PAUL app is not stable enough in all devices and Android versions. Nonetheless, the participants could still experience and engage with the apps and their functionalities and, therefore, provide valuable insights into the apps. Second, the participants were mostly highly educated women (13/20, 65%), which limits our understanding of the perceptions of other potential user groups. Finally, as no control group was used, the quantitative analysis should be interpreted with caution [61].

There are also several strengths to our study. For instance, the dropout rate was relatively low, with 87% (20/23) of the participants completing the study. Furthermore, the app was designed based on theories of change and input from potential end users to increase motivation and capability [41], and the study demonstrated favorable effects. The main study strengths are that the Basic and Smart PAUL apps were tested in a real-life setting and the use of a mixed methods approach to gain insights and a deeper understanding of the feasibility of an intervention.

## Appendix

Multimedia Appendix 1 Description of the Playful Active Urban Living apps.

## Abbreviations

CONSORT-eHEALTH: Consolidated Standards of Reporting Trials of Electronic and Mobile Health Applications and Online Telehealth
cpm: counts per minute
FBM: Fogg Behavior Model
JIT: just-in-time
JITAI: just-in-time adaptive intervention
mHealth: mobile health
MVPA: moderate to vigorous physical activity
PA: physical activity
PAUL: Playful Active Urban Living
