# Supplementary material for: Just-in-Time Prompts for Running, Walking, and Performing Strength Exercises in the Built Environment: 4-Week Randomized Feasibility Study
Source: JMIR Form Res. 2022 Aug 1;6(8):e35268. doi: 10.2196/35268 (PMC9379785; doi:10.2196/35268)
Supplement: Multimedia Appendix 1 [file formative_v6i8e35268_app1.pdf]

Multimedia attachment 1: The Basic and Smart PAUL apps

The PAUL application

The PAUL app aims to increase the recreational walking and/or running time of the user and to motivate the user to perform strength exercises. The app is designed to function as a coach that send you reminders to exercise and supports you during exercise. For this, the app includes five functionalities, namely monitoring, feedback, goal setting, reminder messages and JIT exercise prompts (or exercise prompts).

Mapping the persuasive strategies to the theoretical model

The theoretical assumptions of the PAUL apps are described in detail in an earlier paper (Sporrel K, De Boer RD, Wang S, Nibbeling N, Simons M, Deutekom M, et al. The design and development of a personalized physical activity application based on behavior change principles, incorporating the views of end-users and applying empirical data-mining. Front Public Health. 2020;8:711). Figure 1 provides an overview of these assumptions.

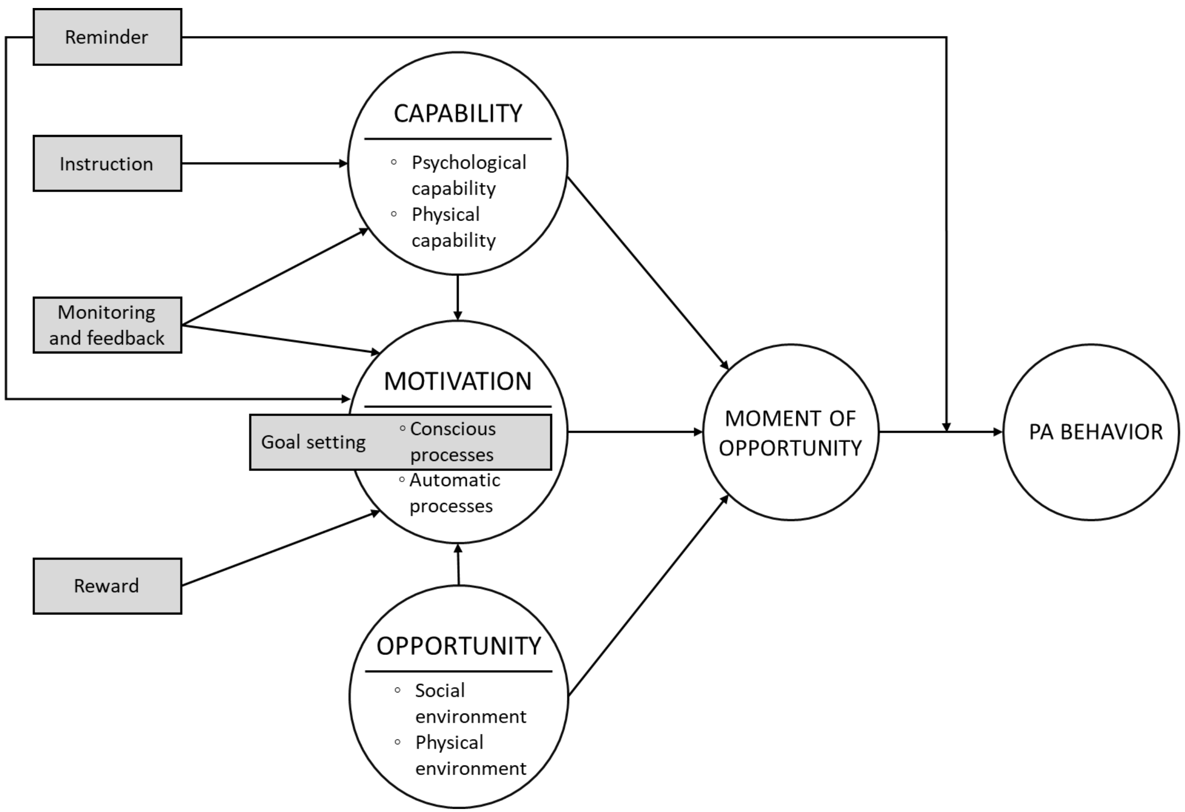

Figure 1: Theoretical assumptions PAUL apps. Published in (Sporrel K, De Boer RD, Wang S, Nibbeling N, Simons M, Deutekom M, et al. The design and development of a personalized physical activity application based on behavior change principles, incorporating the views of end-users and applying empirical data-mining. Front Public Health. 2020;8:711

Design and technical implementation of the functionalities of the PAUL apps

Table 1. Summary of the technical implementation and design characteristics of the functionalities of the PAUL apps.

	JIT exercise prompts	(JITAI) Reminders	Goal setting	Monitoring	Feedback & Praise
Technical implementation					
Delivery system	Smartphone app	Smartphone app	Smartphone app	Smartphone app	Smartphone app

Delivery element	Textual and audio notification, video	Textual and audio notification	Survey, Rapport	GPS, internal clock, survey, data entry field.	Numerical, graphically, rapport, audio.
Frequency	Maximal 3 times per exercise session	Maximal 14 times a week.	On user demand (the user can change her goal when she wants)	On user demand	On user demand
Timing	Tailored, i.e., when the participant passes a GPS location.	Smart PAUL: Tailored, based on the response of the person while using the app, weather, calendar availability and data from the initial training. (but always between 09.00 and 20.00)<br><br>Basic PAUL: randomized	On user demand (the user can change her goal when she wants)	On user demand	On user demand, praise when goal is reached.
Accessibility	The prompt disappears after 30 sec.	System defined. User can decide to keep or delete notification.	The user can always change and view her goal	On user demand	On user demand
Design implementations					
Content	Instruction video. Containing squats or push-ups example with audio support for the pace.	Textual. Three types of messages could be sent: coping messages, feedback messages and affective messages. Messages are positive and affective framed.	Adaptive walking and/or running goal for frequency/week and duration.	-	Feedback on distance crossed, average and current speed, number of strength exercises performed, running route, self-logged behavior outcomes and notes. Textual praise message when reaching the goal on landing page.
Size	30 seconds	Short: 15 to 20 words	-	-	
Intractability	User can ignore, pause, play and stop the video	The user can press ‘read’ or ‘ignore’ or swipe the message away from the notification list of their phone.	The user can fill in a questionnaire to assess baseline fitness, preferred type and frequency of activity and long-term goal.	The user initiates the tracking and can monitor the outcomes of behavior.	None
Complexity	Low (automated by system)	Low (automated by system)	Medium, the participant must perform an initial questionnaire	Low (largely automated by the system)	Low (the feedback is automatically generated by the app)
Tailoring	Location based trigger by system.	Timing is tailored by system. Includes the participants name and is dependent on whether the participant reached their goal.	Based on the input of the participant on fitness level and long-term goal, the app makes a weekly running plan that increases in difficulty when the goal is reached (max 10% per week)	Not tailored.	None.

JIT Exercise prompts

Technical implementation

If the participant is running or walking in Park Transwijk (Utrecht), Sloterplas (Amsterdam) or Oosterpark (Amsterdam) the user is prompted (audio, vibration and pop-up) to perform strength exercises. For each of the parks, around 20 exercise locations were programmed on GPS coordinates. The trigger is sent when the participant passes a GPS point, but only once every five minutes and with a maximum of three per PA session. The user can accept, decline or ignore the prompt.

Design implementation

Two videos (with different exercises) are linked to each exercise location, one video demonstrates squats and the other push ups. When a trigger is activated, the squat or push up was randomly selected. All the instruction videos are recorded at the same location as the GPS points. Therefore, each instruction video is in the same surroundings of the participant. All videos are featuring the same instructor, include a countdown and provides audio cues to guide the participant to perform the exercise at the right speed. Each video lasts about 30 seconds, during which the participant is encouraged to do 10 repetitions of the exercise. The user can view, review, pause and stop whenever she wants. More than 120 different instruction videos of strength exercises were uploaded in the PAUL application.

The screenshot of a trigger to perform a strength exercise is shown in Figure 2. If the app is in foreground, the user receives a pop-up video. If the app is on lock screen, the user receives a pop-up notification of the instruction on which the user can tap to open. The user can choose to watch the video. If the user does not want to watch the video but still wants to perform the exercises, she can monitor her activity by pressing ‘performed’. The video can be displayed on full screen when the phone is tilted to increase visibility of the exercise.

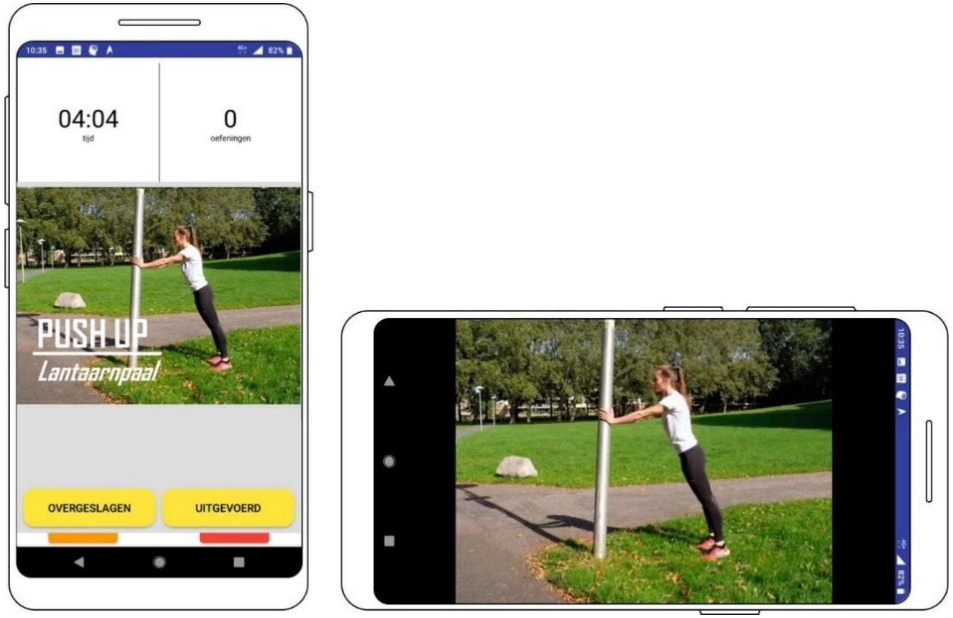

Figure 2: Screenshots of the JIT exercise prompts

(JITAI) Reminders

Technical implementation

When a PAUL notification is sent, an icon will be visible in the phone's status bar and a notification sound will be played. The notification will also be visible on the lock screen of the phone (Figure 3). Users can turn off the reminders in the settings menu of their phone.

The PAUL apps sent fourteen reminder notifications each week between 09.00 and 20.00. Basic PAUL randomly sends a message during that time. Smart PAUL uses a reinforcement learning (RL) module to decide on each decision point (i.e., every hour) whether a reminder would be sent. To make this decision, the RL model uses input from several sources, i.e., the response of the person while using the app, weather, calendar availability and data from the initial training. The initial training of the system was used to give a warm start to the system. For initial training of the module and for finding the important context variables a large dataset of a commercial app was used, containing around 440K runs performed by over 10K users. If the participant reached his/her weekly goal, no reminders were sent for the rest of the week.

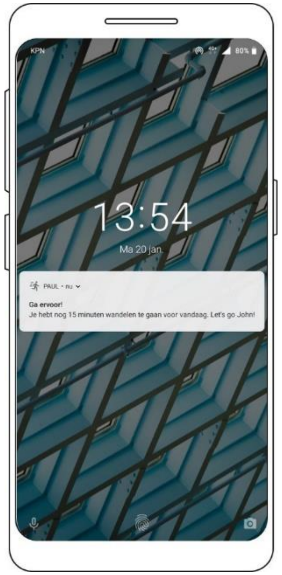

Figure 3: Screenshot of a reminder

Design implementation

When the user receives a message, the user can press 'read' or 'ignore' or swipe the message away from the notification list of their phone. If they click on the notification, the app opens.

The content of the messages was developed for the PAUL apps. They were drafted by 60 applied psychology students and evaluated with survey by 295 Dutch residents. Based on the outcomes of this study, a message library of 141 messages was built. The messages address the individual by name, are positively framed, focus on affective, immediate outcomes and are tailored to the activity type (e.g., running, walking, both). The messages can be classified into three different contents, namely coping, feedback, informative messages (see Table 2). Individuals have 50% change to receive a coping message if they did not reach their goal the week before. In other situations, there is an equal change for receiving a feedback or informative message.

Message type	*Example*
Coping message	Will you go for the extra mile today? You were doing good [*name*]! Don't quit after a setback. Today is a new day.

	Will you go for a run today [*name*]? It can be disappointing when you do not reach your goal. But this happens to the best of us, so keep trying!
Feedback message	How would you feel about going on a walk [*name*]? Today, your goal is to walk [*n*] minutes!<br><br>Are you going to get started to reach your goal today? Still [*n*] minutes to go this week, good luck!
Affective message	Hey [*name*]! Are you going for a walk today? You should ay be proud of yourself when you go!<br><br>Will you make the effort today [*name*]? The most difficult part is to start running. Once you've started, it's fun!

Goal setting

Technical implementation

A goal setting module is implemented in the PAUL apps to set a tailored goal for the user (Figure 4). To set the goal, the user is guided through a short questionnaire (consisting of 5 to 9 questions). Individuals set their goal when they first open the app, but they can change and view her goal when she wants.

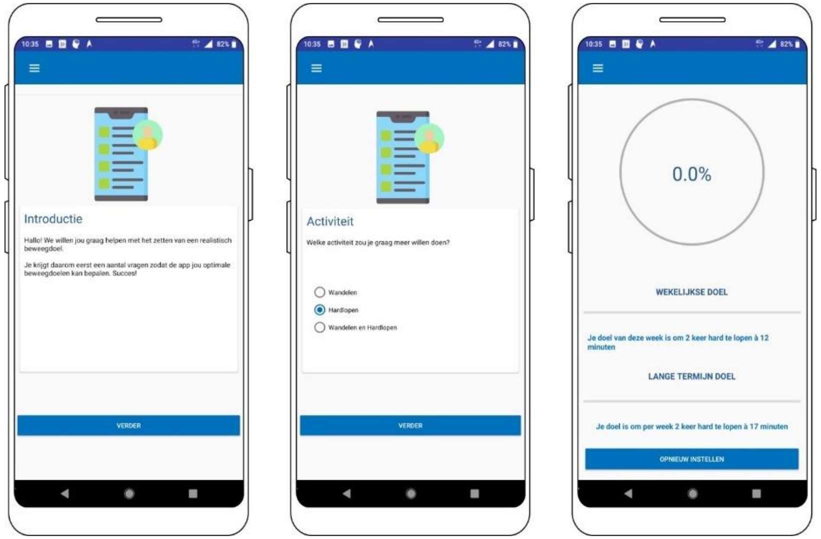

Figure 4: Screenshots of the goal setting module

Design implementation

With the in-app questionnaire the user can choose (1) her preferred activity type (i.e., running, walking or both), (2) her long-term goal (i.e., PA session frequency and duration) and her current fitness level (i.e., current PA frequency and their perceived fitness level). Based on this information, a rule-based system determines a personalized PA program. The duration of the activity increases with approximately 10% per week, but only if the

short-term goal is reached and until the long-term goal is reached. An example of a goal is: to walk two times a week for 20 minutes and run one time per week for 12 minutes.

Monitoring

Technical implementation

Three types of information were monitored with the PAUL app. First, the running and/or walking metrics were recorded automatically by the PAUL app after the user presses 'start' and it stops tracking after the participant presses 'stop'. Time is measured with the internal clock of the participants phone and distance/speed is determined with GPS coordinates. Second, the frequency of performing strength exercises is recorded by the participant by clicking 'performed' or 'skipped' when receiving an exercise prompt. If the participant ignores the prompt, the activity is automatically logged as 'skipped' by the system. Third, the behaviour outcome is recorded by the participant herself by performing a short questionnaire after the run. The participant does not have to fill in the (entire) questionnaire.

Design implementation

The running and/or walking time, distance, speed and route is recorded automatically by the app. The frequency of the performance of strength exercises can be recorded by clicking on the buttons. The behaviour outcomes "how do you feel" can be monitored by performing the 1 to 5 smiley Likert scale. In a similar fashion, participant could rate "how heavy was the exercise" on a five-point Likert scale. Participants could also add notes to their run or walk.

Feedback and praise

Technical implementation

Two types of feedback are provided by the PAUL apps, namely (1) on-screen and audio feedback during physical activity (coined as 'sustained feedback'), and (2) a physical activity rapport (coined as 'cumulative feedback') which can be viewed by the participant immediately after she ended her exercise session and in the history view. Progress towards the weekly goal is visually displayed at the landing page (comparable to a speedometer). The users can (re-)view their PA

rapports as many times and whenever she wants, except during a PA session. Participants receive a textual praise message when reaching the goal on the landing page (Figure 5).

Design implementation

During running or walking, the user can view sustained, numerical feedback on the screen of the app (running or walking duration of the distance crossed, average and current speed, number of strength exercises performed). A simple design ensures that the most important feedback can be viewed with a quick glance. Audio feedback on exercise duration is provided every 5 minutes.

The cumulative feedback consists of the running or walking duration of the distance crossed, the average speed, the number of strength exercises performed and a visualization of their walking or running route. On the landing page, the participant can follow her progress toward her weekly goal.

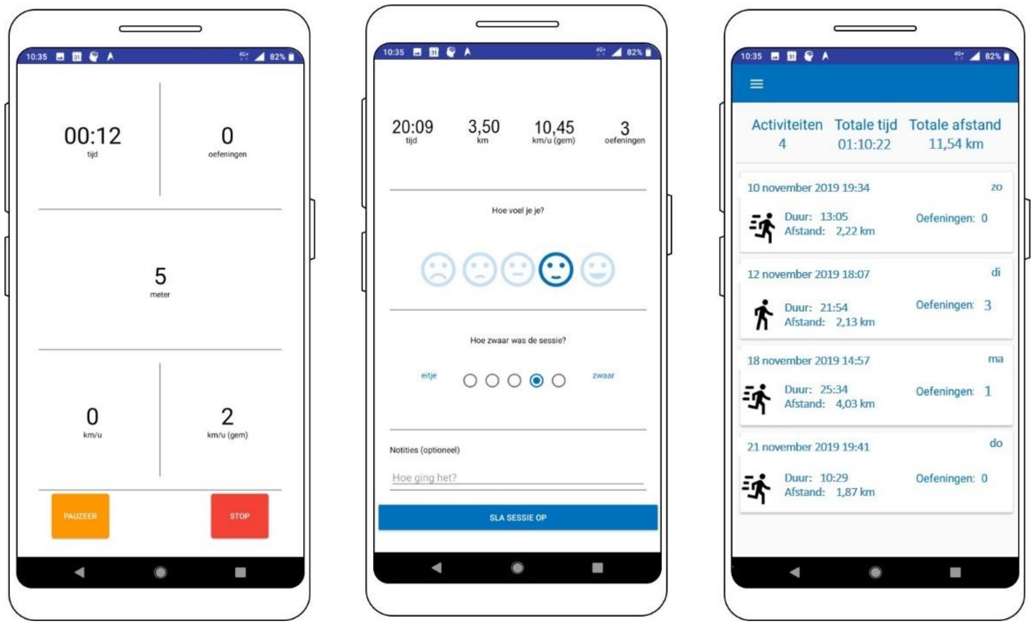

Figure 5: Screenshots of the module feedback
